# Alkaline phosphatase is associated with vascular depression in patients with severe white matter hyperintensities

**DOI:** 10.3389/fnins.2024.1477867

**Published:** 2024-11-25

**Authors:** Xi Tao, Yuqi Yin, Yi Zhang, Chen Yang, Siyuan Wu, Wenjing Tang, Chen Li, Tao Song, Juan He

**Affiliations:** ^1^Department of Neurological Rehabilitation, Hunan Provincial People’s Hospital, Hunan Normal University, Changsha, Hunan, China; ^2^Clinical Research Center for Cerebrovascular Disease Rehabilitation in Hunan Province, Changsha, Hunan, China; ^3^Hunan Provincial Key Laboratory of Neurorestoratology, Hunan Normal University, Changsha, Hunan, China; ^4^Department of Rehabilitation, Rehabilitation Hospital of Hunan Province, Changsha, Hunan, China; ^5^Department of Neurosurgery, Hunan Provincial People’s Hospital, Hunan Normal University, Changsha, Hunan, China

**Keywords:** alkaline phosphatase, association, cerebrovascular disease, vascular depression, white matter hyperintensities

## Abstract

**Background and purpose:**

Cerebrovascular disease (CVD) poses a substantial risk for depression. Elevated levels of alkaline phosphatase (ALP) serve not only as an independent predictive factor for acute cerebrovascular events and unfavorable prognoses but also as a significant predictor of depression in premenopausal women. Nevertheless, the association between elevated ALP levels and vascular depression (VDe) in patients presenting with white matter hyperintensities (WMHs) remains unclear.

**Method:**

In a cross-sectional survey, 265 individuals diagnosed with CVD were incorporated. Baseline demographic information, fasting blood parameters, and MRI data were systematically gathered for analysis. All patients were divided into a severe WMHs (sWMHs) group and a mild WMHs (mWMHs) group based on their Fazekas score. Univariate analysis of potential variables among different subgroups of patients with scores of Hamilton Rating Scale for Depression (HAMD) was performed. Subsequently, the diagnostic effectiveness of multivariables for positive VDe within two WMHs groups was assessed using binary logistic regression. The diagnostic capability of the multivariate approach for VDe was further scrutinized through ordinal logistic regression.

**Results:**

(1) Hypersensitivity C-reactive protein (hs-CRP, *p* = 0.031), high-density lipoprotein cholesterol (HDL-C, *p* = 0.038), apolipoprotein A1 (APOA1, *p* = 0.009), and ALP (*p* = 0.011) exhibited distinct expression in patients with mWMHs across varying HAMD scores. In contrast, erythrocyte counts (*p* = 0.024), hemoglobin (Hb, *p* = 0.011), hs-CRP (*p* = 0.002), and ALP (*p* = 0.021) displayed differential expression in patients with sWMHs across different HAMD scores. (2) ALP and hs-CRP combined with APOA1 or Hb can improve the diagnostic efficiency of positive VDe in sWMHs [AUC = 0.849, 95% CI (0.753, 0.946), *p* < 0.001] or mWMHs [AUC = 0.718, 95% CI (0.603, 0.834), *p* = 0.002] patients, respectively. (3) Alkaline phosphatase (ALP) [OR = 1.016, 95% CI (1.003, 1.028), *p* = 0.016] is correlated with VDe in patients with sWMHs, a relationship that persisted even following adjustments for age and sex.

**Conclusion:**

The amalgamation of multiple markers enhances the diagnostic efficacy of VDe through WMHs classification. Serum ALP is associated with VDe in sWMHs patients.

## 1 Introduction

The concept of vascular depression (VDe) has undergone more than two decades of investigation, encompassing conditions such as cerebral small vessel disease (CSVD) depression, poststroke depression (PSD), and myocardial infarction-related depression (Alexopoulos et al., 1997; Naarding and Beekman, 2011). Criteria grounded in the definition of early clinical symptomatology have facilitated further exploration of VDe (Alexopoulos et al., 1997). González et al. (2012) conducted an epidemiological survey and symptomatic assessment on adults (>50 years old) with cardiovascular or cerebrovascular diseases or significant vascular risk factors, applying DSM-IV criteria. Their findings indicated that 3.4% of patients met the criteria for VDe, with 22.1% of the lifetime depression group classified under the VDe subtype (González et al., 2012). However, the DSM-V does not include diagnostic criteria for VDe. A recent consensus has succinctly delineated and defined the clinical features of VDe and non-VDe in older individuals (≥65 years old), characterized by executive dysfunction, subjective sadness, decreased pleasure, lack of motivation, psychomotor retardation, self-priming deficits, and lack of insight (Aizenstein et al., 2016).

In contrast to clinical symptomatology, which may exhibit overlapping distributions, magnetic resonance imaging (MRI)-defined VDe stands as the second most widely acknowledged criterion and is crucial for establishing a diagnosis of vascular etiology (Krishnan et al., 1997). White matter hyperintensities (WMHs), lacunar infarcts, microbleeds, and macrovascular lesions (such as intracerebral hemorrhage or cerebral infarction) are considered the imaging criteria that must be satisfied for the diagnosis of VDe (Aizenstein et al., 2016; Krishnan et al., 1997). These abnormalities on MRI are well-recognized outcomes of the prolonged impact of vascular risk factors. Damage to blood–brain barrier (BBB) structures, such as microvascular endothelial cells, by vascular risk factors precedes the formation of imaging-recognizable lesions (Kerner and Roose, 2016; Luca and Luca, 2019). Therefore, circulating biomarkers possess an ultra-early predictive value for depression, cognitive impairment, or even schizophrenia, enabling precise prevention of disease or dysfunction (Liu et al., 2022; Wang et al., 2022). However, to our knowledge, apart from PSD, there are scarce reports on biomarkers in systemic or generalized VDe.

Alkaline phosphatase (ALP) serves as a ubiquitous clinical marker for both liver function and bone metabolism, with diverse origins contributing to its presence (Buchet et al., 2013). ALP exhibits tissue specificity in the gut, placenta, and germ cells, whereas in bone, liver, kidney, and brain, ALP possesses characteristics of the tissue-nonspecific alkaline phosphatase (TNAP) isoenzyme (Buchet et al., 2013). Alkaline phosphatase (ALP) emerged as a robust predictor, exhibiting predictive prowess not only for acute ischemic stroke events and cerebral hemorrhage transformation (Kitamura et al., 2022; Liu et al., 2019) but also as an independent prognostic factor for the recurrence of cerebrovascular disease (CVD), adverse functional outcomes, and all-cause mortality in individuals affected by stroke (Zong et al., 2018). In premenopausal women with depression, plasma ALP levels were significantly elevated compared to controls (Petronijević et al., 2008; Cizza et al., 2012). Recently, we observed an association between elevated serum ALP levels and the onset of depression in individuals diagnosed with CVD (Tao et al., 2023). Nevertheless, there is currently no study documenting the potential association between serum ALP levels and depression in patients exhibiting varying degrees of WMHs. A cross-sectional survey was conducted to explore the potential associations among depressive behavior, WMHs and serum ALP in CVD patients.

## 2 Materials and methods

### 2.1 Study participants

A retrospective cross-sectional survey utilizing data from the Department of Neurology and Neurological Rehabilitation at Hunan Provincial People’s Hospital was conducted. All patients signed informed consent forms and voluntarily agreed to participate in questionnaires and blood biomarker tests. For patients who did not have the ability to sign, the forms were signed by family members.

**Figure 1:**
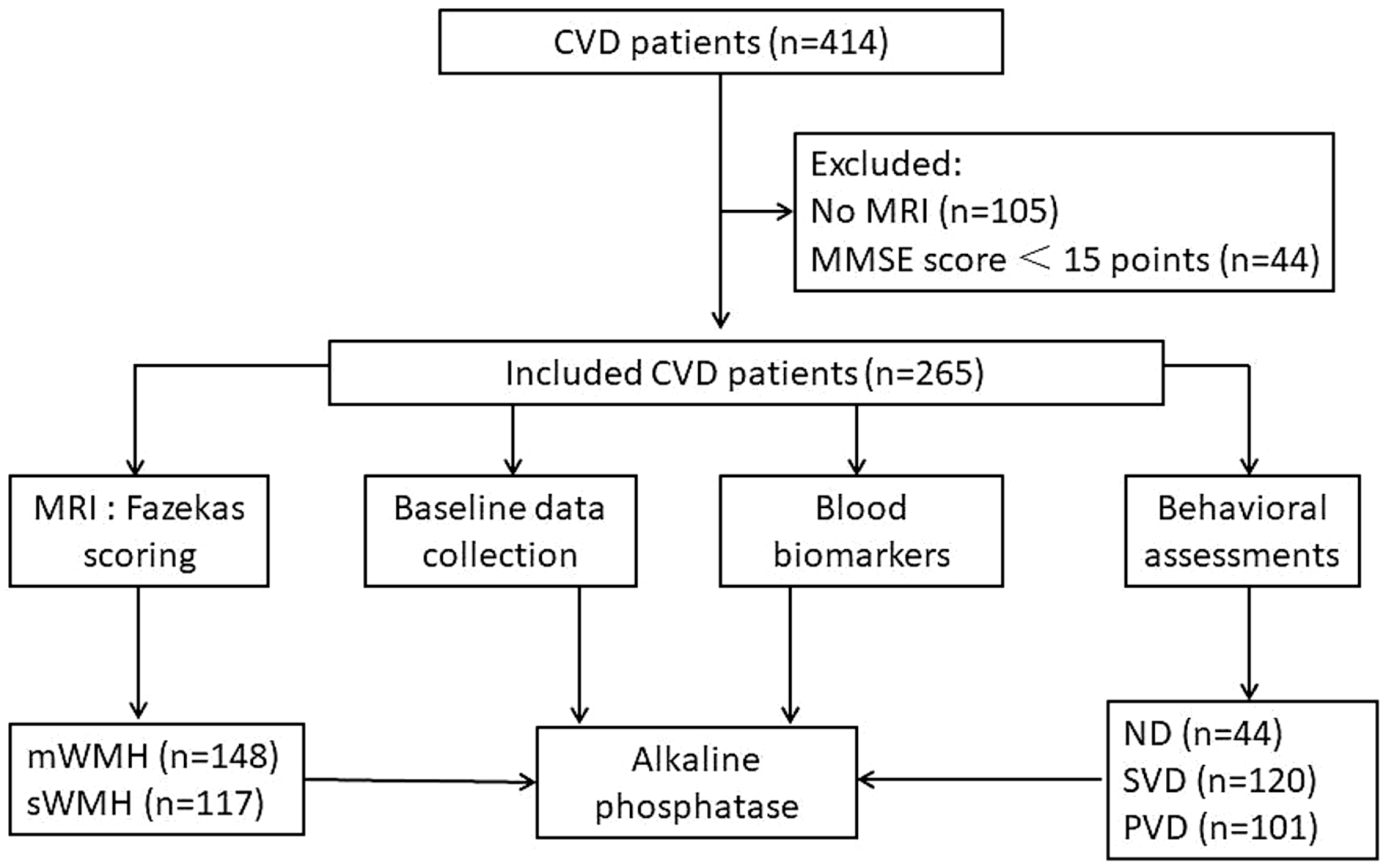
Procedure diagrammatic sketch of research. Data from 414 patients diagnosed with cerebrovascular disease were collected, excluding 105 patients with only CT data and 44 patients with severe cognitive impairment. The final cohort comprised 265 patients. Comprehensive information, neuropsychological scores, MRI data, and blood markers were acquired. Patients were stratified into mWMHs or sWMHs groups based on cumulative Fazekas scores. Subsequently, a diagnostic model for VDe involving alkaline phosphatase was developed.

The demographic information of 414 patients with CVD was gathered between May 2020 and July 2021. Among them, 105 patients had only cranial CT images, and 309 had MRI images. Forty-four patients with Mini Mental State Examination (MMSE) scores less than 15 points were classified as having severe cognitive impairment. Ultimately, 265 patients were included in the study, comprising individuals with stroke (*n* = 203) and CSVD (*n* = 62) (Figure 1).

Patients with CSVD were admitted to the hospital due to various reasons, including anxiety or depression, memory loss, headache, insomnia, dizziness, high blood pressure, or poor glycemic control. Subsequently, brain MRI was performed to assess cerebral atrophy, WMHs, lacunar infarcts, or microbleeds. For individuals with stroke, specific stroke events such as sensory disturbance, limb weakness, dysphagia, or slurred speech were clearly identified. Early cranial CT scans were deemed necessary for patients suspected of having intracranial hemorrhage.

The inclusion criteria were specified as follows: (1) presence of consciousness, (2) stable vital signs, (3) MMSE score greater than 15 points, (4) willingness to participate in the behavioral questionnaire survey, (5) manifestation of relatively stable depressive behavioral symptoms lasting for more than 2 weeks, (6) completion of a routine sequence of brain MRI, and (7) confirmed diagnosis of CSVD or unilateral hemispheric stroke.

The exclusion criteria were delineated as follows: (1) the presence of a clear infection within 2 weeks preceding assessment, (2) severe aphasia, (3) a history indicative of suspected depression prior to the stroke, (4) impairment of glomerular filtration rate [<30 mL/(min × 1.73 m^2^)] or compromised liver function (glutamic pyruvic transaminase >200 U/L), (5) use of antidepressants or benzodiazepines since onset, and (6) incomplete data.

### 2.2 Clinical information

All basic demographic information that may be related to depressive status was collected, including working (employment) status, educational background, body mass index (BMI), sex, age, proportion of CSVD, classification and course of stroke, previous stroke history, smoking, alcohol consumption, diabetes mellitus and coronary atherosclerotic heart disease. A history of stroke denotes the experience of a stroke event leading to hospitalization, with or without subsequent sequelae. Hypertension, diabetes, and coronary heart disease pertain to a well-established diagnosis either before or subsequent to admission. Work (employment) status is divided according to whether or not work and the stability of job.

### 2.3 Behavioral assessment

All behavioral assessments were performed in two phases. The first phase was a cognitive behavioral assessment, and the second phase was followed by a depression and anxiety behavioral assessment for patients with an overall cognitive score that met the requirements (MMSE >15).

We used the MMSE scale to assess global cognitive function in all patients according to the Guidelines of Cognition Classification Consensus Study (Skrobot et al., 2018) and the recommended standards of Vascular Cognitive Impairment (Tao et al., 2022; Tian et al., 2019).

The core symptoms of depression include a lack of sense of worth, low mood, and a diminished interest. Based on the guidelines of the diagnostic requirements, the exclusion criteria from the DSM-IV (Uher et al., 2014) and the Vascular depression consensus report—a critical update (Aizenstein et al., 2016), the diagnosis of VDe has established reference criteria.

The severity of depression was evaluated using the 24-item Hamilton Rating Scale for Depression (HAMD). HAMD scores 8 points indicated no depression (normal); scores ranging from 8 to 20 points indicated suspicion for depression; scores ranging from 20 to 35 points indicated definite depression; and scores above 35 points indicated severe depression. Following this classification, individuals diagnosed with CVD were categorically divided into non-depression (ND), suspicious vascular depression (SVD), and positive vascular depression (PVD) groups or subgroups. For very few patients had severe depression, they were all included in the PVD group. Additionally, the Montgomery Depression Rating Scale (MDRS) was used as a control measure (Montgomery and Asberg, 1979). Given the frequent occurrence of anxiety-depression comorbidities, the Hamilton Rating Scale for Anxiety (HAMA) was employed in this study. Additionally, the modified Barthel Index (MBI) was utilized to assess the activities of daily living in all CVD patients (Qu et al., 2021).

It is essential to clarify that cognitive behavioral assessments were conducted by senior physicians with extensive clinical experience. In cases where patients had MMSE scores exceeding 15 points, the head of the research group initially consulted with the attending physician to gain insights into the patient’s condition and emotional state. Subsequently, skilled physicians, including one leading physician, who had undergone questionnaire training engaged in discussions with the patient and/or family members. Additionally, to gain a more comprehensive understanding of anxiety and depression levels related to illness, sleep, diet, weight, cognition, coping mechanisms, and expectations, the physician, when necessary, liaised with the patient’s physical therapist to assess the patient’s collaboration with rehabilitation training.

### 2.4 Detection of blood biomarkers

All patients fasted for at least 8 h, and blood samples were obtained between 6:00 and 7:00 in the morning. Five milliliters of blood containing procoagulant was collected (HITACHI 7600, Japan) to test for the following biomarkers: alkaline phosphatase (ALP), urea nitrogen, uric acid, homocysteine, cystinase inhibitor C (Cys-C), apolipoprotein (APO) A1, apolipoprotein B, hypersensitive C-reactive protein (hs-CRP), low-density lipoprotein cholesterol (VDL-C), triglycerides, high-density lipoprotein cholesterol (HDL-C) and lipoprotein *α*. Two milliliters of blood anticoagulated was collected by EDTA (XN-10, JAPAN) to perform hematology analysis, including hemoglobin (Hb) concentration and erythrocyte counts. For specific measurement methods of each indicator, refer to our previously published literature (Tao et al., 2023). All indicators were examined using commercial kits by professional operators, and all tests were performed in accordance with the manufacturer’s instructions.

### 2.5 MRI scan and analysis

**Figure 2:**
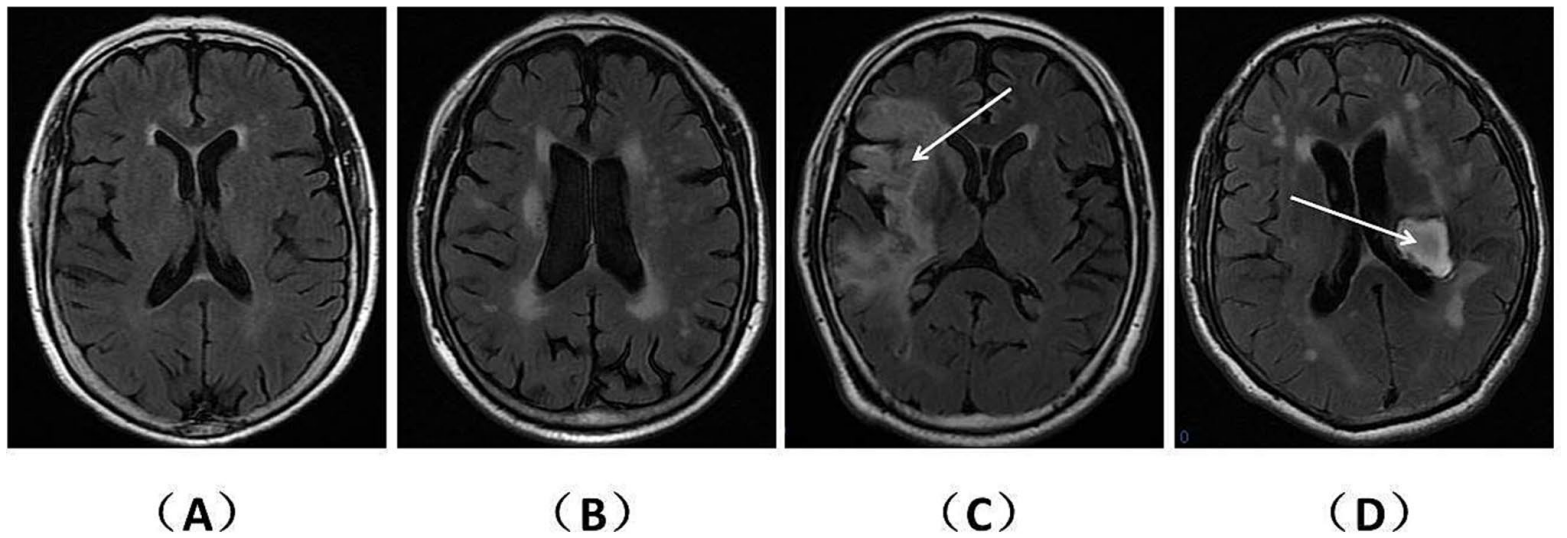
WMHs on representative FLAIR sequences of patients with CSVD and stroke. The T2WI-FLAIR sequences are presented separately for patients with CSVD and stroke. **(A)** (2 points) and **(B)** (5 points) depict the TWMHs scores for different CSVD patients. **(C)** (2 points) and **(D)** (5 points) illustrate the TWMHs scores for patients with ischemic infarction (right hemisphere) and hemorrhage (left basal ganglia), respectively. Notably, panels **(A,C)** meet the criteria for mWMHs, while panels **(B,D)** fulfill the criteria for sWMHs.

T1WI, T2WI, and T2WI-FLAIR sequences from MRI data were acquired using a 1.5-tesla Siemens Trio magnet (Germany). WMHs, encompassing deep WMHs (DWMHs) and periventricular WMHs (PWMHs), were identified as hyperintense areas in the white matter beneath the cerebral cortex on T2WI-FLAIR sequences. Patients with acute or subacute cerebrovascular disease underwent diffusion-weighted imaging to eliminate the influence of lesion edema on WMHs. PWMHs and DWMHs severity were assessed using the Fazekas score (with 0 points indicating regular, 1 point indicating mild, 2 points indicating moderate, and 3 points indicating severe) (Fazekas et al., 1987). In contrast to patients with CSVD, our focus was on identifying WMH features in the unaffected hemisphere of stroke patients. The Fazekas scores for PWMHs and DWMHs on representative FLAIR sequences were calculated. Subsequently, the sum of these scores was determined as the total WMHs (TWMHs) score. Patients with TWMHs scores less than 3 points were categorized as having mild WMHs (mWMHs), while those with TWMHs scores greater than or equal to 3 points were considered to have severe WMHs (sWMHs) (Figure 2).

Furthermore, physicians utilized computer software to aid in the identification of lesions exceeding a diameter of 1.5 cm, encompassing regions such as the cerebellum, brainstem, parietal lobe, frontal lobe, temporal lobe, radiation crowns, basal ganglia, and hippocampus. Two experienced physicians analyzed the neuroimaging data, resolving any disagreements through discussion.

### 2.6 Statistical analysis

(1) The proportions among groups were analyzed by the chi square test. (2) For continuous variables, exploratory analysis was carried out first. If all the data were distributed normally, the mean ± standard deviation (SD) was used; otherwise, the median (interquartile range, IQR) was used. (3) *t*-tests were employed to assess the statistical differences in normally distributed variables between two groups, while Mann–Whitney U tests were utilized for the comparison of non-normally distributed variables. (4) Comparisons among three subgroups were performed using one-way ANOVA if data were normally distributed, followed by Tukey tests. If the homogeneity of variance assumption was not met, Dunnett’s T3 test was used. If any of the variables were nonnormally distributed when comparing three subgroups, the Kruskal–Wallis *H* test was applied, and the Bonferroni correction was used. (5) Within the sWMHs or mWMHs group, the ND population served as the control, and binary logistic regression was employed to assess the diagnostic efficacy of univariate variables (such as ALP, Hb, APOA1, or Hs-CRP) and their amalgamation for PVD. (6) Utilizing Hs-CRP, Hb, APOA1, ALP and as covariates, along with temporal, parietal, frontal, and basal segments (or parietal and basal segments) as factors, the diagnostic efficacy of multivariate analysis for VDe across various WMHs groups was examined through ordinal logistic regression. If the test of parallel lines was not met, multinomial logistic regression was used. SPSS 24.0 statistical software (Chicago, IL, USA) was used to analyze all data.

## 3 Results

### 3.1 Demographic characteristics based on WMHs grouping

Based on the Fazekas score and the aforementioned criteria, two hundred and sixty-five patients were categorized into the sWMHs group (*n* = 117) and mWMHs group (*n* = 148). Within the sWMHs group, no significant differences in demographic characteristics were observed among the three subgroups (Table 1). In the mWMHs group, a progressive increase in the HAMD score corresponded to a gradual decrease in the prevalence of CSVD across different subgroups (*p* = 0.025). However, the smoking rate was different among different subgroups (e.g., it was highest in the SVD group and lowest in the ND group, *p* = 0.014). No discernible differences were observed in educational background, sex, age, prior stroke history, stroke duration, stroke type, diabetes, coronary heart disease, or employment status, alcohol consumption among the three subgroups (Supplementary Table 1).

**Table 1:** Demographic data of patients with different HAMD scores in the sWMHs group.

Variables	ND (*n* = 15)	SVD (*n* = 45)	PVD (*n* = 57)	*Χ^2^/F/H*	*p*
Age, years^a^	65.00 ± 12.08	68.51 ± 10.49	68.84 ± 10.80	0.770	0.465
BMI (kg/m^2^)^a^	23.99 ± 3.94	23.45 ± 3.46	22.82 ± 2.98	0.941	0.393
Sex (male) n (%)	13 (86.67%)	35 (77.78%)	40 (70.18%)	2.112	0.348
Education n (%)					
0	1 (6.67%)	1 (2.22%)	5 (8.77%)	3.226	0.521
<6	3 (20.00%)	6 (13.33%)	11 (19.30%)		
≥7	11 (73.33%)	38 (84.44%)	41 (71.93%)		
Stroke					
Hemorrhagic stroke n (%)	2 (13.33%)	4 (8.89%)	10 (17.54%)	1.391	0.499
Ischemic stroke n (%)	8 (53.33%)	33 (73.33%)	41 (71.93%)		
Disease duration (month) ^b^	12.60 (37.20)	1.15 (7.26)	2.60 (8.00)	3.654	0.161
CSVD n (%)	5 (33.33%)	9 (20.00%)	6 (10.53%)	4.533	0.104
History of stroke n (%)	0 (0.00%)	3 (6.67%)	2 (3.51%)	1.938	0.379
Hypertension n (%)	12 (80.00%)	34 (75.56%)	45 (78.95%)	0.215	0.898
Diabetes mellitus n (%)	6 (40.00%)	15 (33.33%)	23 (40.35%)	0.570	0.752
CHD n (%)	6 (40.00%)	13 (28.89%)	15 (26.32%)	1.032	0.597
Smoking n (%)	8 (53.33%)	19 (42.22%)	17 (29.82%)	3.461	0.177
Alcohol intake n (%)	3 (20.00%)	10 (22.22%)	9 (15.79%)	0.698	0.705
Employment or not n (%)					
Retirement	10 (66.67%)	32 (71.11%)	42 (73.68%)	4.937	0.552
Unemployed	0 (0.00%)	2 (4.44%)	0 (0.00%)		
Liberal professions	1 (6.67%)	4 (8.89%)	6 (10.53%)		
Stable operation	4 (26.67%)	7 (15.56%)	9 (15.79%)		

a Expressed as the mean ± SD.

b Expressed as the median (IQR).

### 3.2 Comparison of lesion site counts and brain atrophy between the mWMHs and sWMHs groups

We assessed the presence of each lesion exceeding 1.5 cm in diameter and evaluated brain atrophy using MRI information. Classification was based on the severity of WMHs. Within the mWMHs group, the proportion of responsible lesions in the basal ganglia (*p* = 0.027), temporal lobe (*p* = 0.004), frontal lobe (*p* = 0.036), and parietal lobe (*p* = 0.006) gradually increased with the severity of depression across the three subgroups. Conversely, in the sWMHs group, the difference observed with the severity of depression was limited to an increase in the percentage of responsible lesions in the basal ganglia (*p* = 0.047) and parietal lobe (*p* = 0.020). No significant differences were found in the proportion of other lesions among the distinct subgroups (Table 2).

**Table 2:** Comparison of the lesion site counts and brain atrophy among different subgroups.

Variables	Groups	ND	SVD	PVD	*χ* ^2^	*p*
Brain atrophy	mWMHs	21 (72.41%)	43 (57.33%)	33 (75.00%)	4.587	0.101
	sWMHs	15 (100%)	40 (88.89%)	52 (91.23%)	3.033	0.220
Frontal lobe	mWMHs	1 (3.45%)	14 (18.67%)	12 (27.27%)	6.670	**0.036** ^*^
	sWMHs	2 (13.33%)	7 (15.56%)	15 (26.32%)	2.356	0.308
Parietal lobe	mWMHs	0 (0.00%)	12 (16.00%)	9 (20.45%)	10.347	**0.006** ^**^
	sWMHs	0 (0.00%)	5 (11.11%)	13 (22.81%)	7.856	**0.020** ^*^
Temporal lobe	mWMHs	0 (0.00%)	11 (14.67%)	10 (22.73%)	11.184	**0.004** ^**^
	sWMHs	2 (13.33%)	6 (13.33%)	8 (14.04%)	0.012	0.994
Occipital lobe	mWMHs	3 (10.34%)	9 (12.00%)	7 (15.91%)	0.567	0.753
	sWMHs	1 (6.67%)	6 (13.33%)	3 (5.26%)	2.117	0.347
Insular lobe	mWMHs	0 (0.00%)	4 (5.33%)	5 (11.36%)	5.452	0.065
	sWMHs	1 (6.67%)	2 (4.44%)	2 (3.51%)	0.270	0.874
Thalamus	mWMHs	1 (3.45%)	4 (5.33%)	3 (6.82%)	0.408	0.816
	sWMHs	2 (13.33%)	7 (15.56%)	10 (17.54%)	0.184	0.912
Cerebellum	mWMHs	2 (6.90%)	5 (6.67%)	3 (6.82%)	0.002	0.999
	sWMHs	1 (2.27%)	3 (6.67%)	3 (5.26%)	0.103	0.950
Hippocampus	mWMHs	2 (6.90%)	2 (2.67%)	1 (2.27%)	1.162	0.559
	sWMHs	0 (0.00%)	0 (0.00%)	0 (0.00%)	/	/
Brain stem	mWMHs	4 (13.79%)	12 (16.00%)	7 (15.91%)	0.086	0.958
	sWMHs	5 (33.33%)	10 (22.22%)	14 (24.56%)	0.714	0.700
Basal ganglia	mWMHs	4 (13.79%)	18 (24.00%)	18 (40.91%)	7.223	**0.027** ^*^
	sWMHs	2 (13.33%)	14 (31.11%)	26 (45.61%)	6.106	**0.047** ^*^
Lateral ventricle	mWMHs	1 (3.45%)	1 (1.33%)	4 (9.09%)	4.090	0.129
	sWMHs	1 (2.27%)	4 (8.89%)	8 (14.04%)	1.044	0.593
Corona radiata	mWMHs	3 (10.34%)	12 (16.00%)	5 (11.36%)	0.828	0.661
	sWMHs	2 (13.33%)	3 (6.67%)	7 (12.28%)	1.093	0.579
Centrum ovale	mWMHs	0 (0.00%)	3 (4.00%)	1 (2.27%)	2.041	0.360
	sWMHs	1 (2.27%)	2 (4.44%)	5 (8.77%)	0.768	0.681
Corpus callosum	mWMHs	0 (0.00%)	2 (2.67%)	3 (6.82%)	3.359	0.186
	sWMHs	0 (0.00%)	2 (4.44%)	3 (5.26%)	1.441	0.487
Subtentorial Stroke	mWMHs	5 (17.24%)	15 (20.00%)	10 (22.73%)	0.332	0.847
	sWMHs	5 (33.33%)	11 (24.44%)	13 (22.81%)	0.675	0.714

^*^p < 0.05, ^**^p < 0.01.

### 3.3 Comparison of blood markers and behavioral scores between the mWMHs and sWMHs groups

Compared with the mWMHs group, the sWMHs had higher β2-M levels (*p* = 0.004), Cys-C levels (*p* = 0.041), older age (*p* < 0.001), higher BMI (*p* < 0.001), higher PWMHs scores (*p* < 0.001), DWMHs scores (*p* < 0.001), TWMHs scores (*p* < 0.001), HAMD scores (*p* = 0.001) and MDRS scores (*p* < 0.001). Additionally, the sWMHs group had lower BMI (*p* = 0.019), MMSE scores (*p* < 0.001) and MBI scores (*p* = 0.001). The comparison of other blood markers or HAMA scores between the two groups showed no obvious differences (Table 3).

**Table 3:** Multivariable comparison between the sWMHs and mWMHs groups.

Variables	sWMHs (*n* = 117)	mWMHs (*n* = 148)	*F/U*	*p*
Age, years^a^	68.00 (16.50)	62.00 (17.00)	5.192	**<0.001** ^***^
BMI (kg/m^2^)^b^	23.21 ± 3.29	24.11 ± 2.79	4.627	**0.019** ^*^
Erythrocyte (×10^12^/L)^a^	4.25 (0.72)	4.24 (0.82)	0.717	0.473
Hb (g/L)^a^	129.00 (22.50)	130.00 (22.75)	1.224	0.221
hs-CRP (mg/L)^a^	2.31 (5.19)	1.83 (3.66)	1.058	0.290
Homocysteine (μmol/L)^a^	15.00 (5.97)	13.96 (6.13)	1.574	0.116
Urea nitrogen (mmol/L)^a^	5.15 (1.67)	5.32 (2.13)	1.654	0.098
Uric acid (μmol/L)^b^	331.79 ± 96.77	344.15 ± 94.93	0.785	0.298
Cys-C (mg/L)^a^	1.11 (0.26)	1.04 (0.28)	2.042	**0.041** ^*^
ALP (U/L)^a^	71.00 (24.00)	68.00 (21.75)	0.946	0.344
LDL-C (mmol/L)^a^	2.28 (1.12)	2.44 (1.44)	1.417	0.156
HDL-C (mmol/L)^a^	1.01 (0.41)	1.05 (0.38)	0.852	0.394
Triglycerides (mmol/L)^a^	1.26 (0.79)	1.39 (0.89)	1.875	0.061
Lipoprotein *α* (mg/L)^a^	149.40 (243.79)	141.62 (223.28)	0.503	0.615
APOA1 (g/L)^a^	1.04 (0.28)	1.08 (0.30)	0.594	0.553
APOB (g/L)^a^	0.67 (0.28)	0.72 (0.34)	1.437	0.151
MMSE^a^	25.00 (6.00)	28.00 (4.00)	4.351	**<0.001** ^***^
MBI^a^	80.00 (45.00)	90.00 (43.75)	3.253	**0.001** ^**^
HAMA^a^	10.00 (6.00)	9.00 (5.75)	1.165	0.244
HAMD^a^	19.00 (12.00)	13.00 (11.00)	3.419	**0.001** ^**^
MDRS^a^	14.00 (10.00)	10.00 (8.00)	3.750	**<0.001** ^***^

a Expressed as the mean ± SD.

b Expressed as the median (IQR).

^*^p < 0.05, ^**^p < 0.01, ^***^p < 0.001.

### 3.4 Comparison of blood biomarkers and behavioral scores among subgroups based on WMHs grouping

In the mWMHs group, the levels of hs-CRP (*p* = 0.031) (Supplementary Figure 1A), ALP (*p* = 0.011) (Supplementary Figure 1B), HDL-C (*p* = 0.038) and APOA1 (*p* = 0.009) (Supplementary Figure 1C) were significantly different among the three subgroups. Compared with the ND group, the expression of ALP (*p* = 0.008) in the SVD subgroup was increased, while the expression of HDL-C (*p* = 0.032) was decreased; the expression of hs-CRP (*p* = 0.026) in the PVD subgroup was increased, while APOA1 levels were decreased in both the SVD (*p* = 0.003) and PVD (*p* = 0.043) subgroups. Behavioral assessment showed that MMSE (*p* = 0.001) and MBI (*p* < 0.001) scores decreased with increasing depression scores. Compared with the ND subgroup, the MBI score was reduced in both the SVD (*p* = 0.041) and PVD (*p* < 0.001) subgroups, while the MMSE score (*p* = 0.007) was decreased only in the PVD subgroup (Supplementary Table 2).

**Figure 3:**
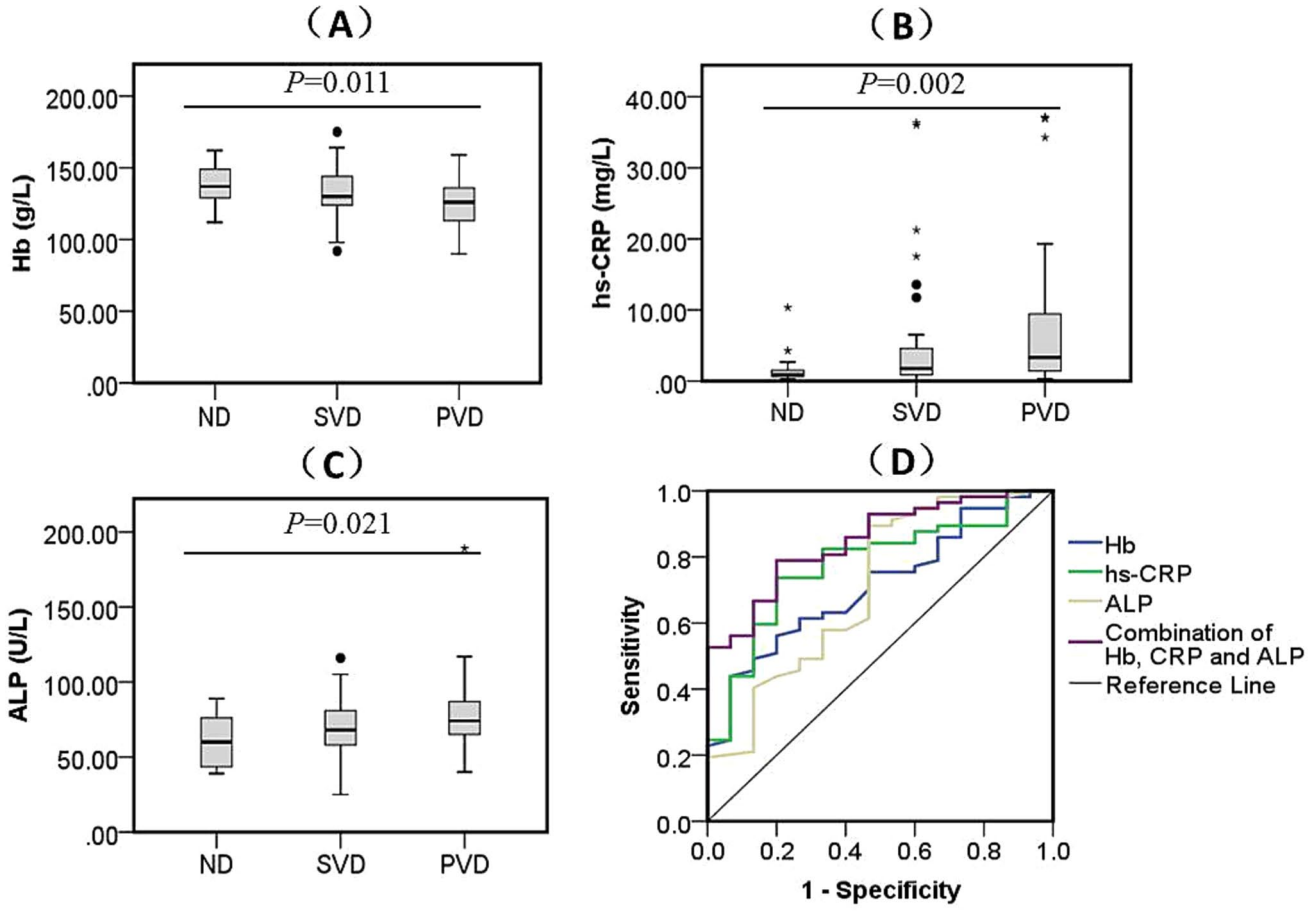
Comparison of biomarkers levels among three subgroups and the diagnostic efficacy for PVD in sWMHs patients. The contrasts in Hb **(A)** (*F* = 4.723, *p* = 0.011), hs-CRP **(B)** (*H* = 12.821, *p* = 0.002), and ALP **(C)** (*F* = 7.756, *p* = 0.021) across different HAMD scores are depicted. Additionally, their amalgamation **(D)** [AUC = 0.849, 95% CI (0.753, 0.946), *p* < 0.001] was utilized for the diagnosis of PVD in the sWMHs group. The black dots represent outliers, and the “*” symbol denotes extreme values.

In the sWMHs group, the erythrocyte count (*p* = 0.024) and the concentrations of Hb (Figure 3A, *p* = 0.011), hs-CRP (Figure 3B, *p* = 0.002) and ALP (Figure 3C, *p* = 0.021) were markedly different among the three subgroups. The erythrocyte count (*p* = 0.046) in the SVD subgroup was lower than that in the ND subgroup, whereas in the PVD subgroup, there was a decline in the erythrocyte count (*p* = 0.006) and Hb level (*p* = 0.008) as well as an increase in hs-CRP (*p* = 0.003) and ALP levels (*p* = 0.033). In addition, the PVD subgroup had lower Hb levels (*p* = 0.028) than the SVD subgroup. Behavioral assessment showed that the MMSE (*p* = 0.003) and MBI (*p* < 0.001) scores decreased gradually with increasing depression scores among the three subgroups. The MMSE (*p* = 0.007) and MBI (*p* = 0.011) scores of the PVD subgroup were obviously lower than those of the SVD subgroup. The MBI score was lower in the other two subgroups (*P*_SVD_ = 0.006, *P*_PVD_ < 0.001) than in the ND subgroup (Table 4).

**Table 4:** Comparison of blood biomarkers and behavioral scores among the three subgroups with sWMHs.

Variables	ND (*n* = 15)	SVD (*n* = 45)	PVD (*n* = 57)	*H/F*	*p* value	Tukey/adjusted by Bonferroni		
						ND vs. SVD	ND vs. PVD	SVD vs. PVD
Erythrocyte (× 10^12^/L)^a^	4.59 ± 0.51	4.29 ± 0.52	4.18 ± 0.50	3.867	**0.024** ^*^	**0.046** ^*^	**0.006** ^**^	0.311
Hb (g/L)^a^	138.07 ± 13.86	132.44 ± 15.95	125.00 ± 17.95	4.723	**0.011** ^*^	0.262	**0.008** ^**^	**0.028** ^*^
hs-CRP (mg/L)^b^	0.87 (0.97)	1.77 (3.82)	3.32 (8.66)	12.821	**0.002** ^**^	0.254	**0.003** ^**^	0.070
Homocysteine (μmol/L)^b^	15.61 (8.40)	15.00 (6.32)	14.68 (5.49)	0.166	0.920	/	/	/
Urea nitrogen (mmol/L)^b^	5.19 (1.66)	5.23 (1.52)	4.85 (2.04)	1.688	0.430	/	/	/
Uric acid (μmol/L)^b^	344.00 (159.00)	315.00 (157.00)	322.00 (105.50)	1.555	0.460	/	/	/
Cys-C (mg/L)^b^	1.13 (0.30)	1.11 (0.25)	1.11 (0.31)	0.715	0.699	/	/	/
ALP (U/L)^b^	60.00 (34.00)	68.00 (23.00)	74.00 (23.00)	7.756	**0.021** ^*^	0.661	**0.033** ^*^	0.188
LDL-C (mmol/L)^b^	2.63 (0.92)	2.11 (1.37)	2.28 (1.06)	3.265	0.195	/	/	/
HDL-C (mmol/L)^b^	0.95 (0.64)	1.08 (0.46)	0.97 (0.37)	1.615	0.446	/	/	/
Triglycerides (mmol/L)^b^	1.79 (1.61)	1.29 (0.87)	1.22 (0.67)	2.618	0.270	/	/	/
Lipoprotein α (mg/L)^b^	88.38 (206.20)	172.62 (259.93)	149.95 (249.94)	3.702	0.157	/	/	/
APOA1 (g/L)^b^	1.02 (0.13)	1.05 (0.34)	1.04 (0.28)	0.000	1.000	/	/	/
APOB (g/L)^b^	0.74 (0.20)	0.64 (0.36)	0.66 (0.24)	1.878	0.391	/	/	/
TWMHs^b^	4.00 (2.00)	3.00 (1.50)	4.00 (2.00)	1.905	0.386	/	/	/
PWMHs^b^	2.00 (1.00)	2.00 (1.00)	2.00 (1.00)	3.189	0.203	/	/	/
DWMHs^b^	2.00 (1.00)	2.00 (1.00)	2.00 (1.00)	0.250	0.882	/	/	/
MMSE^b^	27.00 (5.00)	27.00 (4.50)	23.00 (6.00)	11.459	**0.003** ^**^	1.000	0.064	**0.007** ^**^
MBI^b^	100.00 (0.00)	85.00 (40.00)	60.00 (40.00)	28.973	**<0.001** ^***^	**0.006**	**<0.001** ^***^	**0.011** ^*^
HAMA^b^	4.00 (6.00)	9.00 (4.00)	13.00 (5.00)	41.958	**<0.001** ^***^	**0.026**	**<0.001** ^***^	**<0.001** ^***^
HAMD^b^	5.00 (2.00)	14.00 (5.50)	24.00 (7.00)	95.914	**<0.001** ^***^	**0.009**	**<0.001** ^***^	**<0.001** ^***^
MDRS^a^	4.00 ± 1.96	11.09 ± 3.72	19.33 ± 4.79	102.238	**<0.001** ^***^	**0.005**	**<0.001** ^***^	**<0.001** ^***^

a Expressed as the mean ± SD.

b Expressed as the median (IQR).

^*^p < 0.05, ^**^p < 0.01, ^***^p < 0.001.

### 3.5 Diagnostic efficacy of multivariable combination in PVD

In the mWMHs group, the ROC curves were plotted using PVD as a diagnostic target. We found that hs-CRP is a strong related factor for depression in CVD patients [AUC = 0.662, 95% CI (0.537, 0.787), *p* = 0.020]; the cutoff value was 5.820 mg/L, with a specificity of 93.10% and sensitivity of 38.64%. ALP was a related factor for PVD [AUC = 0.635, 95% CI (0.507, 0.763), *p* = 0.052]; the cutoff value was 81.00 U/L, with a specificity of 96.55% and sensitivity of 27.27%. APOA1 was also an important factor [AUC = 0.655, 95% CI (0.532, 0.779), *p* = 0.026]; the cutoff value was 1.017 g/L, with a specificity of 43.18% and sensitivity of 93.10%. Collectively, the incorporation of hs-CRP, ALP, and APOA1 markedly enhanced the diagnostic effectiveness for PVD [AUC = 0.718, 95% CI (0.603, 0.834), *p* = 0.002]; the cutoff was 0.607, with a specificity of 75.86% and sensitivity of 65.91% (Supplementary Figure 1D).

In the sWMHs group, the ROC curves were plotted using the PVD as a diagnostic target. We found that Hb had a protective effect on depression in patients with CVD [AUC = 0.713, 95% CI (0.580, 0.846), *p* = 0.012]; the cutoff was 122.00 g/L, with a specificity of 43.86% and sensitivity of 93.33%. Similarly, hs-CRP and ALP were found to be important related factors for PVD in this group of patients. For hs-CRP, the AUC was 0.776 [95% CI (0.652, 0.900), *p* = 0.001], with a cutoff of 1.63 mg/L, exhibiting a specificity of 80.00% and sensitivity of 73.68%. Regarding ALP, the AUC was 0.709 [95% CI (0.551, 0.868), *p* = 0.013], and the cutoff was 60.50 U/L, showing a specificity of 53.33% and sensitivity of 89.47%. Furthermore, the incorporation of Hb, hs-CRP, and ALP significantly enhanced the diagnostic efficacy for PVD in this group [AUC = 0.849, 95% CI (0.753, 0.946), *p* < 0.001], with a cutoff of 0.744, specificity of 80.00%, and sensitivity of 78.95% (Figure 3D).

### 3.6 Multivariate analysis with ordinal logistic regression in VDe

Taking into account the diagnostic potential of various blood markers in the mWMHs and sWMHs groups, we incorporated four continuous variables (Hb, hs-CRP, ALP, and APOA1) into the regression model to investigate their diagnostic efficacy for VDe.

In the mWMHs group, four categorical variables (basal ganglia, parietal lobe, frontal lobe, and temporal lobe) were incorporated into Model 1. The initial model demonstrated an acceptable goodness of fit (*p* = 0.578); however, tests of parallel lines revealed an unusual pattern (*χ*^2^ = 21.875, *p* = 0.005), indicating a lack of satisfaction with the proportional odds assumption. Consequently, multinomial logistic regression was chosen. The subsequent results indicated an acceptable goodness of fit (χ^2^ = 261.318, *p* = 0.699), and the model fitting information indicated its superiority to the constant-term model (*p* < 0.001). Using the ND subgroup as a reference, evaluation models for SVD and PVD were constructed. Parameter estimates revealed that APOA1 (*p* = 0.017) and ALP (*p* = 0.011) were diagnostic factors for SVD. However, the four blood markers showed no diagnostic value for PVD. APOA1 (*p* = 0.019) and ALP (*p* = 0.025) were identified as independent diagnostic factors for SVD, even after adjusting for the proportion of CSVD, sex, and age (Supplementary Table 3).

In the sWMHs group, two categorical variables (parietal lobe and basal ganglia) were included in the model, and four continuous variables (Hb, hs-CRP, ALP, and APOA1) were integrated to construct an ordinal regression model. The results of Model 2 demonstrated an acceptable goodness of fit (*χ*^2^ = 194.434, *p* = 0.909), with fit indices surpassing those of the model featuring only the constant term (*p* < 0.001). The test of parallel lines affirmed the existence of the proportional odds assumption (*χ*^2^ = 4.376, *p* = 0.626). Parameter estimates highlighted that Hb (*p* = 0.029) and ALP (*p* = 0.013) were pivotal diagnostic factors for VDe. Even after adjusting for sex and age, Model 2 maintained an acceptable goodness of fit (*χ*^2^ = 192.564, *p* = 0.909), with superior fit indices compared to the model with only the constant term (*p* < 0.001). The test of parallel lines indicated the continued existence of the proportional odds assumption (*χ*^2^ = 5.489, *p* = 0.704). Parameter estimates revealed that ALP (*p* = 0.016) remained an independent diagnostic factor for VDe (Table 5).

**Table 5:** Multivariate diagnosis of VDe in sWMHs patients by ordinal logistic regression.

Variables	OR (95% CI)	*p*	^#^Adjusted OR (95% CI)	*p*
Basal ganglia	1.656 (1.032, 2.656)	**0.036** ^*^	0.828 (1.065, 2.775)	**0.027** ^*^
Parietal lobe	1.927 (0.968, 3.836)	0.062	1.719 (0.993, 3.980)	0.052
Hb	0.985 (0.971, 0.998)	**0.029** ^*^	0.987 (0.973, 1.002)	0.096
hs-CRP	1.016 (0.992, 1.039)	0.187	1.013 (0.990, 1.037)	0.262
ALP	1.015 (1.003, 1.028)	**0.013** ^*^	1.016 (1.003, 1.028)	**0.016** ^*^
APOA1	0.996 (0.367, 2.701)	0.994	0.759 (0.242, 2.385)	0.637

# Shows sex and age adjusted. ^*^p < 0.05.

## 4 Discussion

Our investigation unveiled several novel insights: (1) In mWMHs patients, hs-CRP, HDL-C, APOA1, and ALP exhibited differential expression, while in those with sWMHs, erythrocyte counts, Hb, hs-CRP, and ALP showed differential expression, particularly in correlation with varying HAMD scores. (2) The combination of ALP and hs-CRP with APOA1 or Hb demonstrated enhanced diagnostic efficacy for VDe in mWMHs or sWMHs patients, respectively. (3) ALP is associated with the development of VDe in patients with sWMHs.

The identification of peripheral-specific blood markers serves dual roles in both the diagnosis and prognosis of PSD, and this approach can be expanded to encompass a broader spectrum of CVDs, including CSVD or stroke combined with CSVD. In contrast to univariate models, multivariate detection methods have the capability to elucidate the biological mechanisms underlying VDe through the exploration of multiple pathological pathways, thereby enhancing diagnostic efficacy (Sun et al., 2022).

WMHs represent a notable imaging feature in patients with CSVD (Guo et al., 2021). Many elderly individuals experiencing stroke events exhibit extensive cerebral microvascular disease before the onset of strokes. Consequently, considering WMHs as a reference standard for assessing the severity of cerebral small vessel injury becomes particularly pertinent for further analysis of the potential value of blood markers in VDe. Our study identified ALP and hs-CRP as common prospective markers of depression in both WMHs populations, suggesting that nonspecific inflammatory responses and elevated ALP levels, with unclear functions, may contribute to the formation and development of VDe. Hs-CRP, a nonspecific inflammatory marker produced by hepatocytes, has been noted to be significantly elevated in patients with PSD compared to those without PSD (Mu et al., 2018). Early elevation of hs-CRP in patients with ischemic stroke independently predicts the occurrence of PSD 6 months after the stroke (Yang et al., 2016). Our study supports the association of depressive behavior in CVD patients with the body’s nonspecific inflammatory response, consistent with previous findings (Yang et al., 2016). Similarly, ALP, commonly used as a clinical indicator for abnormal liver function, has demonstrated predictive value for acute CVD and prognosis in various studies (Kitamura et al., 2022; Liu et al., 2019; Zong et al., 2018). Notably, it has been identified as one of the bone metabolic characteristics associated with depression in premenopausal women (Cizza et al., 2012). In the nervous system, TNAP, an isoform of ALP, mediates cerebral vascular smooth muscle cell calcification and neurotransmitter synthesis. Elevated TNAP expression in the hippocampus and serum has been linked to cognitive disorders in Alzheimer’s Disease patients (Buchet et al., 2013; Vardy et al., 2012). Our study is the first to reveal that serum ALP concentrations in patients with CVD increase with rising depression scores.

Nonetheless, this study identified a statistically significant reduction in HDL-C and APOA1 exclusively within the mWMHs group. In contrast, the reduction in erythrocytes and Hb was observed solely in the sWMHs group. This discrepancy suggests that the pathophysiological mechanisms underlying the occurrence of VDe may vary across different degrees of WMHs. APOA1, a fundamental protein constituent of HDL-C, holds essential roles in cholesterol reverse transport, while also exhibiting antioxidant, anti-inflammatory, and antithrombotic functions, promoting endothelial cell repair and microRNA transport (van der Vorst, 2020; Lu et al., 2022). A previous meta-analysis established a significant association between low APOA1 expression and an increased incidence of depression (Bot et al., 2020). Plasma HDL-C levels were markedly decreased in patients with PSD compared to non-PSD patients, although changes in APOA1 were not statistically significant (Shen et al., 2019). Moreover, an electroshock regimen targeting specific types of depression demonstrated that improvements in depressive behavior were correlated with increased APOA1 levels (Aksay et al., 2016). In our study, APOA1 levels were diminished in both PVD and SVD patients compared with nondepressed subjects, aligning with previous reports (Bot et al., 2020; Shen et al., 2019; Aksay et al., 2016). A cross-sectional study involving 8,640 middle-aged and elderly volunteers (50–75 years old) in the community revealed a clear correlation between anemia and depressive behavior (Vulser et al., 2020). In a cohort study involving young adults (mean age 38.4 years) without chronic diseases or medication history, depressive participants exhibited lower hemoglobin concentrations than their nondepressive counterparts, and the prevalence of anemia exhibited a significant dose–response relationship with the severity of depression (Vulser et al., 2016). Furthermore, recent research demonstrated that postpartum anemia significantly increased the risk of postpartum depression (Maeda et al., 2020). These findings collectively suggest distinct roles for Hb, erythrocytes, HDL-C, and APOA1 in the development of VDe based on WMHs classification.

Additionally, we conducted an analysis of blood markers in both the mWMHs and sWMHs groups, revealing a significant elevation solely in Cys-C in the latter group. This finding aligns with previous reports (Zhang et al., 2019). Intriguingly, we did not observe any differences in ALP, hs-CRP, Hb, or APOA1, consistent with our prior findings (Tao et al., 2022).

There is a prevailing academic consensus emphasizing the enhanced diagnostic efficacy achieved through the amalgamation of multiple markers. In this study, we focused on PVD as the diagnostic target. By integrating the findings of univariate analysis, we discovered that the combination of hs-CRP, ALP, and APOA1 significantly improved the diagnostic efficacy of VDe in patients with mWMHs. Notably, in the sWMHs group, the diagnostic efficacy, encompassing AUC, specificity, and sensitivity, of the combination involving Hb, hs-CRP, and ALP surpassed that of the mWMHs group. This outcome underscores the advantages of employing a combined diagnostic approach with multiple markers reflecting diverse pathological characteristics, thereby establishing a foundation for potential clinical applications.

To explore the potential diagnostic significance of four blood markers in predicting depression occurrence among patients with CVD, we employed ordinal logistic regression. Considering the distinct severities of WMHs, we initially conducted a univariate analysis of demographic information and lesion distribution among patients with varying HAMD scores in the two groups. Subsequently, variables showing differential distribution in the covariates were incorporated into subsequent model construction. Surprisingly, in the mWMHs group, Model 1 failed to meet the proportional odds assumption, and only ALP and APOA1 emerged as independent diagnostic factors for SVD, even after adjusting for confounding factors. This suggests that, in the mWMHs population, investigations into PVD may need to extend beyond biological factors to encompass the impact of non-biological factors such as social psychology, environment, and gender. Contrastingly, in the sWMHs group, the same four blood markers were integrated into Model 2, with corrections made for differences in the distribution of lesions in the parietal lobe and basal ganglia. Results demonstrated that Model 2 exhibited excellent diagnostic value. ALP (*p* = 0.016) emerged as an independent associated factor for depression in CVD patients with sWMHs, even after adjusting for age and sex.

Plasma ALP originates from various sources, including bones, liver, intestine, and the brain (Buchet et al., 2013). In premenopausal patients with depression, elevated plasma ALP levels coincide with abnormal bone metabolism (Petronijević et al., 2008). While the role of intestinal and liver-derived ALP in depression pathogenesis remains unexplored, TNAP abnormalities play a crucial role in the calcification of intracranial vascular smooth muscle cells and atherosclerosis formation (Buchet et al., 2013; Ren et al., 2022). In chronic inflammatory conditions, hs-CRP and inflammatory cytokines collaborate with ALP to promote vascular calcification (Liu et al., 2012; Haarhaus et al., 2022). The human and rodent cerebral cortex exhibit higher TNAP levels compared to other brain regions (Fonta et al., 2015). In ALP knockout mice, the vitamin B6-dependent intracellular microenvironment is compromised, leading to decreased glutamate decarboxylase activity and serotonin levels (Narisawa et al., 2001; Waymire et al., 1995). In a sepsis mouse model, TNAP mediates damage to microvascular endothelial cells, resulting in increased BBB permeability, reduced spontaneous movement, and diminished interest in spatial exploration (Nwafor and Brown, 2021). Lipopolysaccharide, commonly used in bacterial infection models, induces systemic inflammation involving vascular neural units and is employed in rodents to model depression (Zhao et al., 2019). TNAP has been found to inhibit the binding of Lipopolysaccharide to Toll-like Receptor 4 ligands, thereby mitigating the inflammatory response (Graser and Liedtke, 2021). Subsequent studies revealed a negative correlation between elevated TNAP expression and activity with cognitive function in the serum and hippocampus of Alzheimer’s Disease patients (Vardy et al., 2012; Pike et al., 2015). Under chronic inflammatory stimuli, TNAP translocates from the cell membrane anchoring site to the circulation. Functionally transformed TNAP not only exhibits a weak anti-inflammatory mechanism but, more importantly, unveils the molecular basis of BBB structure and function (Pike et al., 2015). Therefore, we hypothesize that ALP or TNAP might be implicated in the pathophysiology of VDe by mediating BBB injury.

The study has certain limitations, including: (1) a relatively small sample size of CVD patients, especially patients without depression; (2) imprecise quantification of white matter degeneration volume; and (3) data collection from individual centers, with a scarcity of patients from community settings. Prospective comprehensive mood screening, the implementation of multicenter longitudinal cohort studies, and more precise quantification of WMHs in high-risk community-based CVD patients could enhance the interpretability of the findings.

## 5 Conclusion

Our discoveries propose that Hb, hs-CRP, ALP, and APOA1 could serve as potential hematologic biomarkers for diagnosing VDe in patients with WMHs. Furthermore, our findings emphasize the potential diagnostic enhancement achieved by examining multiple factors concurrently. Notably, among these factors, ALP stood out as an independent variable associated with VDe in patients with sWMHs.

## Data Availability

The raw data supporting the conclusions of this article will be made available by the authors, without undue reservation.

## Supplementary material

SUPPLEMENTARY FIGURE 1 Comparison of biomarkers levels among three subgroups and the diagnostic efficacy for PVD in mWMHs patients. The contrasts in hs-CRP **(A)** (*F* = 6.921, *p* = 0.031), ALP **(B)** (*H* = 8.946, *p* = 0.011), and APOA1 **(C)** (*F* = 4.847, *p* = 0.009) across different HAMD scores are depicted. Additionally, their amalgamation **(D)** [AUC = 0.718, 95% CI (0.603, 0.834), *p* = 0.002] was utilized for the diagnosis of PVD in the mWMHs group.
