# Supplementary material for: Alkaline phosphatase is associated with vascular depression in patients with severe white matter hyperintensities
Source: Front Neurosci. 2024 Nov 25;18:1477867. doi: 10.3389/fnins.2024.1477867 (PMC11625733; doi:10.3389/fnins.2024.1477867)
Supplement: Supplementary file 1 [file Table_1.DOCX]

| **Variable** | | | **ND (n = 29)** | **SVD (n =75)** | | **PVD (n=44)** | | ***Χ^2^/F/H*** | ***P*** | | |
| --- | --- | --- | --- | --- | --- | --- | --- | --- | --- | --- | --- |
| Age (years) ^a^ | | | 62.62±10.71 | 58.60±13.27 | | 60.39±12.73 | | 1.099 | 0.336 | | |
| BMI (kg/m^2^) ^a^ | | | 23.90±2.75 | 24.40±2.95 | | 23.76±2.53 | | 0.830 | 0.438 | | |
| Sex (male) n (%) | | | 17 (58.62%) | 52(69.33%) | | 27(61.36%) | | 1.390 | 0.499 | | |
| Education (years) n (%) | | |  |  | |  | |  |  | | |
|  | 0 | | 0 (0.00%) | | 0 (0.00%) | | 0 (0.00%) | 0.608 | | 0.738 |  |
|  | <6 | | 6 (20.69%) | | 11 (14.67%) | | 8 (18.18%) |  |  |  |  |
|  | ≥7 | | 23 (79.31%) | | 64 (85.33%) | | 36 (81.82%) |  |  |  |  |
| Stroke | | |  |  | |  | |  |  | | |
|  | | Hemorrhagic stroke n (%) | 2 (6.90%) | 6 (8.00%) | | 10 (22.73%) | | 4.310 | 0.116 | | |
|  |  | Ischemic stroke n (%) | 14 (48.28%) | 50 (66.67%) | | 27 (61.36%) | |  |  |  |  |
|  |  | Disease duration (month) ^b^ | 3.50 (10.27) | 1.20 (2.45) | | 1.50 (3.27) | | 2.812 | 0.245 | | |
| CSVD n (%) | | | 14 (48.28%) | 19 (65.52%) | | 9 (20.45%) | | 7.350 | **0.025^*^** | | |
| History of stroke n (%) | | | 1 (3.45%) | 1 (1.33%) | | 2 (4.55%) | | 1.185 | 0.553 | | |
| Hypertension n (%) | | | 23 (79.31%) | 56 (74.67%) | | 37 (84.09%) | | 1.472 | 0.479 | | |
| Diabetes mellitus n (%) | | | 11 (37.93%) | 30 (40.00%) | | 15 (34.09%) | | 0.412 | 0.814 | | |
| CHD n (%) | | | 5 (17.24%) | 14 (18.67%) | | 13 (29.55%) | | 2.345 | 0.310 | | |
| Smoking n (%) | | | 3 (10.34%) | 30 (40.00%) | | 15 (34.09%) | | 8.471 | **0.014^*^** | | |
| Alcohol intake n (%) | | | 6 (20.69%) | 12 (16.00%) | | 9 (20.45%) | | 0.514 | 0.773 | | |
| Employment or not n (%) | | |  |  | |  | |  |  | | |
|  | | Retirement | 16 (55.17%) | 28 (37.33%) | | 16 (36.36%) | | 6.528 | 0.367 | | |
|  | | Unemployed | 1 (3.45%) | 2 (2.67%) | | 1 (2.27%) | |  |  |  |  |
|  | | Liberal professions | 2 (6.90%) | 17 (22.67%) | | 12 (27.27%) | |  |  |  |  |
|  | | Stable operation | 10 (34.48%) | 28 (37.33%) | | 15 (34.09%) | |  |  |  |  |

**Supplementary Table 1 Basic demographic data of patients with different HAMD scores in the mWMHs group**

^a^ expressed as the mean ± SD. ^b^ expressed as the median (IQR). ^*^*P*˂0.05.

**Supplementary Table 2 Comparison of biomarkers and behavioral scores among the three subgroups with mWMHs**

| **Variables** | **ND (n = 29)** | **SVD (n = 75)** | **PVD (n = 44)** | ***F / H*** | ***P* value** | **Tukey / adjusted by Bonferroni** | | |
| --- | --- | --- | --- | --- | --- | --- | --- | --- |
|  |  |  |  |  |  | **ND *vs.* SVD** | **ND *vs.* PVD** | **SVD *vs.* PVD** |
| Erythrocyte (× 10^12^/L) ^a^ | 4.22 (0.59) | 4.27 (0.82) | 4.19 (0.85) | 0.801 | 0.670 | / | / | / |
| Hb (g/L) ^a^ | 129.00 (20.00) | 133.00 (22.00) | 128.00 (25.00) | 0.388 | 0.824 | / | / | / |
| hs-CRP (mg/L) ^a^ | 1.11 (2.73) | 1.79 (2.46) | 3.12 (7.24) | 6.921 | **0.031^*^** | 0.285 | **0.026^*^** | 0.495 |
| Homocysteine (μmol/L) ^a^ | 12.53 (3.90) | 14.08 (8.26) | 14.04 (5.30) | 3.442 | 0.179 | / | / | / |
| UN (mmol/L) ^a^ | 5.71 (1.54) | 5.17 (2.22) | 5.31 (2.78) | 0.723 | 0.697 | / | / | / |
| UA (μmol/L) ^b^ | 358.68 ± 84.13 | 335.37 ± 90.45 | 349.55 ± 108.67 | 0.728 | 0.484 | 0.264 | 0.689 | 0.434 |
| Cys-C (mg/L) ^a^ | 1.04 (0.35) | 1.04 (0.29) | 1.05 (0.23) | 0.643 | 0.725 | / | / | / |
| ALP (U/L) ^a^ | 62.00 (20.50) | 72.00 (26.00) | 68.00 (23.75) | 8.946 | **0.011^*^** | **0.008^**^** | 0.131 | 1.000 |
| TG(mmol/L) ^a^ | 1.54 (1.08) | 1.37 (0.83) | 1.34 (0.93) | 0.309 | 0.857 | / | / | / |
| HDL-C (mmol/L) ^a^ | 1.10 (0.36) | 1.00 (0.34) | 1.13 (0.47) | 6.515 | **0.038^*^** | **0.032^*^** | 0.299 | 1.000 |
| LDL-C (mmol/L) ^b^ | 2.75 ± 0.96 | 2.50 ± 0.96 | 2.47 ± 1.00 | 0.823 | 0.441 | / | / | / |
| LPa (mg/L) ^a^ | 104.07 (304.59) | 169.43 (222.71) | 133.53 (204.51) | 2.222 | 0.329 | / | / | / |
| APOA1 (g/L) ^b^ | 1.19 ± 0.14 | 1.06 ± 0.21 | 1.08 ± 0.23 | 4.847 | **0.009^**^** | **0.003^**^** | **0.043^*^** | 1.000 |
| APOB (g/L) ^a^ | 0.80 (0.44) | 0.72 (0.35) | 0.70 (0.28) | 1.072 | 0.585 | / | / | / |
| TWMHs ^a^ | 2.00 (1.00) | 1.00 (2.00) | 1.00 (1.00) | 5.197 | 0.074 | / | / | / |
| PWMHs ^a^ | 1.00 (0.50) | 1.00 (1.00) | 1.00 (0.00) | 2.800 | 0.247 | / | / | / |
| DWMHs ^a^ | 1.00 (1.00) | 0.00 (1.00) | 0.00 (1.00) | 7.870 | **0.020^*^** | **0.017^*^** | 0.093 | 1.000 |
| MMSE ^a^ | 29.00 (3.00) | 28.00 (3.00) | 25.00 (5.00) | 14.514 | **0.001^**^** | 1.000 | **0.007^**^** | **0.001^**^** |
| MBI ^a^ | 100.00 (7.50) | 95.00 (35.00) | 57.50 (50.00) | 35.308 | **0.000^***^** | **0.041^*^** | **0.000^***^** | **0.000^***^** |
| HAMA ^a^ | 4.00 (4.50) | 9.00 (4.00) | 14.00 (6.75) | 62.985 | **0.000^***^** | **0.000^***^** | **0.000^***^** | **0.000^***^** |
| HAMD ^a^ | 5.00 (3.00) | 12.00 (5.00) | 24.50 (8.75) | 123.129 | **0.000^***^** | **0.000^***^** | **0.000^***^** | **0.000^***^** |
| MDRS ^a^ | 4.00 (3.50) | 9.00 (5.00) | 18.00 (6.75) | 104.449 | **0.000^***^** | **0.000^***^** | **0.000^***^** | **0.000^***^** |

^a^ expressed as the mean ± SD. ^b^ expressed as the median (IQR). ^*^*P*˂0.05, ^**^ *P*˂0.01, ^***^ *P*˂0.001.

**Supplementary Table 3 Multivariate diagnosis of VDe in mWMHs patients by multinomial logistic regression**

| **Variables** | | **OR (95% CI)** | ***P*** | **^#^Adjusted OR (95% CI)** | ***P*** |
| --- | --- | --- | --- | --- | --- |
| SVD | Frontal lobe | / | / | 1.065 (0.092, 12.298) | 0.960 |
|  | Parietal lobe | / | / | / | / |
|  | Temporal lobe | / | / | / | / |
|  | Basal ganglia | 1.178 (0.308, 4.500) | 0.810 | 1.135 (0.264, 4.876) | 0.864 |
|  | Hb | 0.988 (0.959, 1.018) | 0.427 | 0.992 (0.960, 1.024) | 0.609 |
|  | hs-CRP | 0.993 (0.863, 1.144) | 0.927 | 0.995 (0.860, 1.151) | 0.944 |
|  | ALP | 1.048 (1.011, 1.087) | **0.011** | 1.044 (1.005, 1.083) | **0.025** |
|  | APOA1 | 0.030 (0.002, 0.534) | **0.017** | 0.019 (0.001, 0.525) | **0.019** |
| PVD | Frontal lobe | / | / | 1.436 (0.115, 17.935) | 0.779 |
|  | Parietal lobe | / | / | / | / |
|  | Temporal lobe | / | / | / | / |
|  | Basal ganglia | 3.022 (0.756, 12.088) | 0.118 | 3.044 (0.648, 14.298) | 0.158 |
|  | Hb | 0.987 (0.954, 1.021) | 0.439 | 0.996 (0.960, 1.033) | 0.812 |
|  | hs-CRP | 1.093 (0.949, 1.259) | 0.219 | 1.091 (0.942, 1.264) | 0.245 |
|  | ALP | 1.035 (0.997, 1.076) | 0.074 | 1.032 (0.993, 1.074) | 0.112 |
|  | APOA1 | 0.153 (0.007, 3.502) | 0.240 | 0.054 (0.002, 1.867) | 0.106 |

^#^ shows the proportion of CSVD, age, and sex adjusted. ^*^*P*˂0.05.
