# Supplementary figures and images for: Alkaline phosphatase is associated with vascular depression in patients with severe white matter hyperintensities
Source: Front Neurosci. 2024 Nov 25;18:1477867. doi: 10.3389/fnins.2024.1477867 (PMC11625733; doi:10.3389/fnins.2024.1477867)

## Slide 1
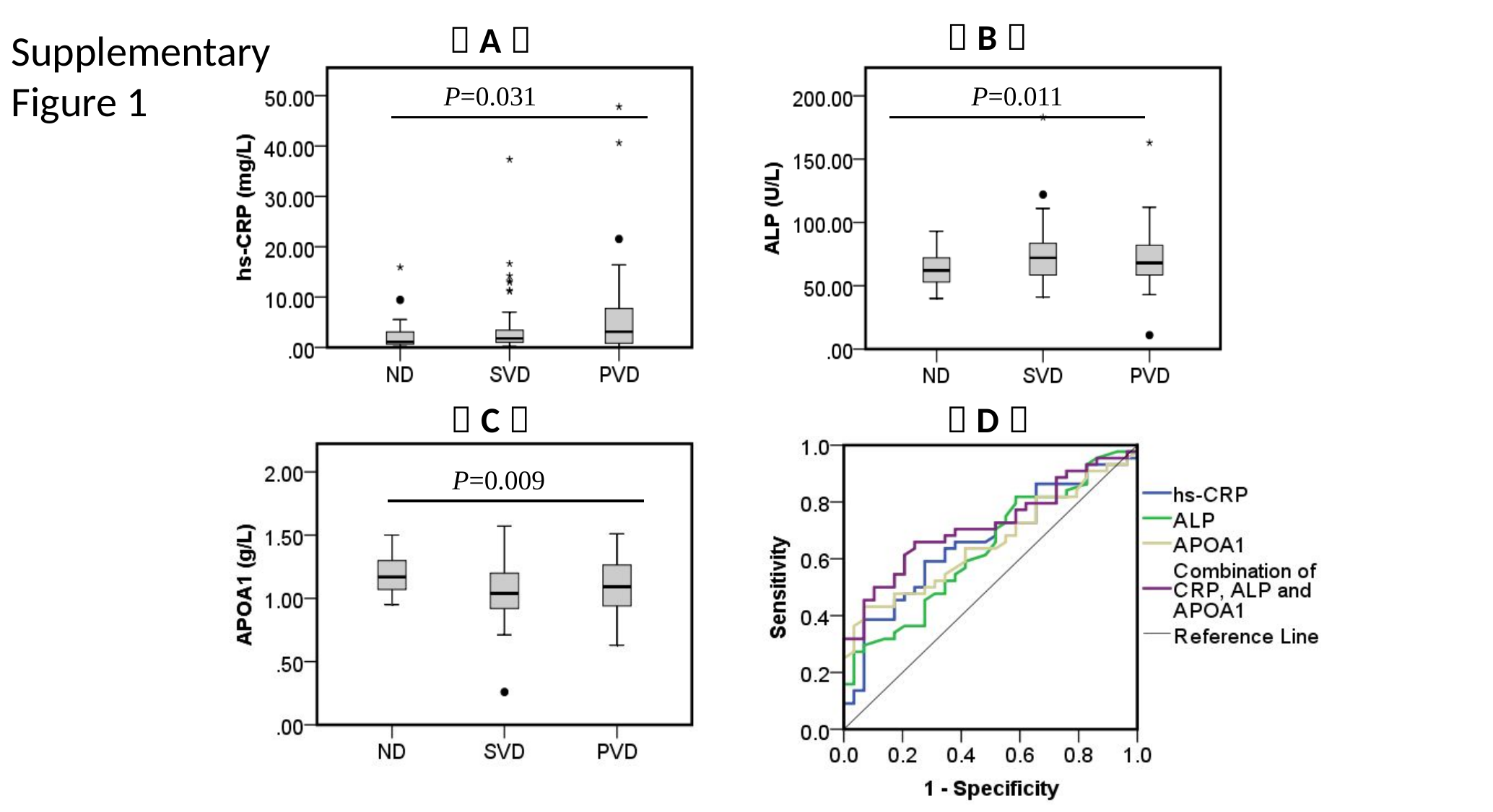

（B）
（A）
Supplementary Figure 1
P=0.031
P=0.011
（C）
（D）
P=0.009

Supplement: SUPPLEMENTARY FIGURE 1 — Comparison of biomarkers levels among three subgroups and the diagnostic efficacy for PVD in mWMHs patients. The contrasts in hs-CRP (A) (F = 6.921, p = 0.031), ALP (B) (H = 8.946, p = 0.011), and APOA1 (C) (F = 4.847, p = 0.009) across different HAMD scores are depicted. Additionally, their amalgamation (D) [AUC = 0.718, 95% CI (0.603, 0.834), p = 0.002] was utilized for the diagnosis of PVD in the mWMHs group. [file Presentation_1.PPTX]
